# Ultra-Stable Potassium Ion Storage of Nitrogen-Doped Carbon Nanofiber Derived from Bacterial Cellulose

**DOI:** 10.3390/nano11051130

**Published:** 2021-04-27

**Authors:** Liang Ma, Jinliang Li, Zhibin Li, Yingying Ji, Wenjie Mai, Hao Wang

**Affiliations:** 1 Guangdong Provincial Key Laboratory of Micro/Nano Optomechatronics Engineering, College of Mechatronics and Control Engineering, Shenzhen University, Shenzhen 518060, China; maliang2415@jnu.edu.cn; 2 Siyuan Laboratory, Guangdong Provincial Engineering Technology Research Center of Vacuum Coating Technologies and New Materials, Department of Physics, Jinan University, Guangzhou 510632, China; lijinliang@email.jnu.edu.cn (J.L.); pibetaguita@gmail.com (Z.L.); jiyingying07@163.com (Y.J.); wenjiemai@email.jnu.edu.cn (W.M.)

**Keywords:** nitrogen doping, carbon nanofiber, bacterial cellulose, relative energy density, potassium ion batteries

## Abstract

As a promising energy storage system, potassium (K) ion batteries (KIBs) have received extensive attention due to the abundance of potassium resource in the Earth’s crust and the similar properties of K to Li. However, the electrode always presents poor stability for K-ion storage due to the large radius of K-ions. In our work, we develop a nitrogen-doped carbon nanofiber (N-CNF) derived from bacterial cellulose by a simple pyrolysis process, which allows ultra-stable K-ion storage. Even at a large current density of 1 A g^−1^, our electrode exhibits a reversible specific capacity of 81 mAh g^−1^ after 3000 cycles for KIBs, with a capacity retention ratio of 71%. To investigate the electrochemical enhancement performance of our N-CNF, we provide the calculation results according to density functional theory, demonstrating that nitrogen doping in carbon is in favor of the K-ion adsorption during the potassiation process. This behavior will contribute to the enhancement of electrochemical performance for KIBs. In addition, our electrode exhibits a low voltage plateau during the potassiation–depotassiation process. To further evaluate this performance, we calculate the “relative energy density” for comparison. The results illustrate that our electrode presents a high “relative energy density”, indicating that our N-CNF is a promising anode material for KIBs.

## 1. Introduction

Due to the high energy density and high conversion efficiency, lithium-ion batteries (LIBs) have dominated most energy storage systems in portable electronic devices [1,2,3,4,5]. With the rapid development of green energy sources, further development of large-scale energy storage systems is urgently needed [6,7]. However, the shortage of lithium resources greatly limits the further use of LIBs in the large-scale energy storage field. It is necessary to further develop alternative energy storage technologies with low costs. Compared with LIBs, potassium ion batteries (KIBs) gradually entered the field of scientists’ view due to the abundance of potassium (K) resource in the Earth’s crust and the low electrode potential (K/K^+^ = 2.93 V vs. SHE) and are considered to be one of the most promising alternatives to replace LIBs in large-scale energy storage systems [8,9,10]. However, due to the large radius of the K-ion, the development of appropriate anode materials for K-ion storage is still a significant challenge.

In recent years, extensive efforts have been put into developing anode materials for KIBs, including carbonaceous materials, metals, oxides and sulfides [11,12,13,14]. In consideration of the economy and sustainability, carbonaceous materials are deemed as the first choice of anode in KIBs [15,16,17]. Of all the carbonaceous materials, graphite (a conventional anode material in LIBs) was first investigated. The results have proved that graphite presented a high specific capacity for K-ion storage [18,19,20]. However, this high-performance of graphite in KIBs only exists in extremely low current densities, which severely restricted their further practical application. To address this issue, scientists began to explore non-graphitic carbon materials for KIBs [21,22]. As one of the non-graphitic carbon materials, hard carbon has gradually received great attention due to its natural “pseudo-graphite” structure [23,24,25]. Moreover, the dual storage mechanism of inner-layer intercalation and near-surface absorption also accelerates the kinetic process of hard carbon for K-ion storage, which facilitates its better performance rate [26,27]. Up to now, a great deal of work involving hard carbon for K-ion storage has been reported [28,29]. As one kind of hard carbon, the carbon nanofiber derived from bacterial cellulose has received lots of attention, which has been widely applied in supercapacitors, capacitive deionization, LIBs and NIBs [30,31,32,33,34,35,36]. Therefore, the carbon nanofiber derived from bacterial cellulose was also considered for application in KIBs [37]. However, the hard carbons derived from bacterial cellulose still cannot meet the demand of KIBs. Further improvements to the K-ion storage performance are still highly desired. According to the previous reports, nitrogen doping in hard carbon could remarkably enhance its specific capacity for KIBs [38,39,40]. Nevertheless, these works cannot balance the high specific capacity, high rate and ultra-long cycling life required for K-ion storage. Further developing nitrogen-doped hard carbon with these three important parameters for KIBs is still a great challenge.

In this work, we develop a nitrogen-doped carbon nanofiber (N-CNF) derived from bacterial cellulose using a one-step pyrolysis process, which presents ultra-stable K-ion storage. Even at a large current density of 1 A g^−1^, our electrode presents a reversible specific capacity of 81 mAh g^−1^ after 3000 cycles for KIB, with a capacity retention ratio of 71%. To investigate the electrochemical enhancement performance of our N-CNF, we provide the calculation results according to density functional theory (DFT), demonstrating that nitrogen doping in carbon is in favor of K atom adsorption during the potassiation process. This behavior will contribute to the enhancement of the electrochemical performance for KIBs. In addition, we noticed that our electrode exhibited a low voltage plateau during the potassiation–depotassiation process. This behavior is helpful for the improvement of energy density in a full K-ion battery. To further evaluate this performance, we calculate the “relative energy density” of our N-CNF for comparison, and the result illustrates that our electrode presents a high “relative energy density”, which indicates that our N-CNF is a promising anode material for KIBs.

## 2. Experimental

### 2.1. Synthesis

The bacterial cellulose was obtained from Hainan Yeguo Foods Co. Ltd. (Haikou, China) for the preparation of N-CNF; the bacterial cellulose was firstly frozen and then transferred into a bulk tray dryer for freeze-drying (temperature <40 °C, pressure <20 Pa) for 48 h to obtain the dried cellulose. Subsequently, the 0.5 g dried cellulose and 0.5 g urea were separately put in a tube furnace and heat-treated at 800 °C with a heating rate of 5 °C min^−1^ in flowing Ar, and the N-CNF was obtained. For comparison, the carbon nanofiber without nitrogen doping was synthesized by the same procedure without the addition of urea, which was named CNF.

### 2.2. Characterization

The morphologies of our samples were tested by field-emission scanning electron microscope (SEM, Zeiss Ultra 55, Jena, Germany) and high-resolution transmission electron microscopy (TEM, JEOL-2100). The elemental mapping was obtained by TEM (JEOL-2100), equipped with an Oxford Aztec energy dispersive spectrometer. The structures were recorded by X-ray diffraction (XRD, Rigaku MiniFlex 600, Osaka, Japan) using Cu K-Alpha radiation (λ = 0.15406 nm) and Raman spectrometry (Horiba T64000, Kyoto, Japan) using a laser with a wavelength of 532 nm. The surface properties were confirmed by X-ray photoelectron spectroscopies, which were measured using an image photoelectron spectrometer (Thermo Fisher Scientific K-Alpha, Waltham, MA, USA) with Al K-Alpha X-ray source. The specific surface area was calculated by nitrogen adsorption isotherms according to the Brunauer–Emmett–Teller (BET) method, which was obtained by a nitrogen adsorption apparatus (×1000; Biaode-Kubo, Beijing, China) at 77 K.

### 2.3. Computational Methods

The first-principles calculations were carried out based on DFT as implemented in the Vienna Ab-initio Simulation Package (VASP). The Perdew–Becke–Ernzerhof (PBE) functional and projector-augmented wave (PAW) schemes were adopted for geometric optimizations. A total of 500 eV was used for the energy cut-off throughout the calculations. The adsorption energy (Δ*E_a_*) was defined as the following equation: Δ*E_a_ = E*_2_ *− E*_1_ *− µ_K_*, where *E*_1_ and *E*_2_ were the total energies of the system before and after adsorbing potassium ions, respectively, and *µ_K_* was the chemical potential of a single K atom.

### 2.4. Electrochemical Measurement

For the preparation of the electrode, active material, carbon black and carboxymethyl cellulose with the ratio of 8:1:1 in moderate water were mixed into a sizing. Subsequently, the obtained sizing was blade-coated on the rough Cu foil, dried and cut into a disc with a diameter of 14 mm for the electrode in the KIB. The CR2032-type half battery was assembled into a glovebox (Etelux Lab2000, Beijing, China) filled with argon. Metallic K foil and Whatman fiber filter were adopted as the counter electrode and separator, respectively. A total of 0.1 mL KPF_6_ in ethylene carbonate/propylene carbonate (1:1, *v*/*v*) mixed solvent was applied to an electrolyte solution. The galvanostatic charge–discharge (GCD) curves and cycle performances were recorded by a battery test system (Neware BTS-4000, Shenzhen, China) at 50 mA g^−1^ unless otherwise noted. Cyclic voltammetry (CV) curves were performed via an electrochemical workstation (Chenhua C1030, Shanghai, China) at a scan rate of 0.2 mV s^−1^ except as otherwise noted. Electrochemical impedance spectroscopy (EIS) was carried out via another electrochemical workstation (Princeton STAT-3400, Berwyn, USA) with a frequency range of 0.1 Hz–100 kHz.

## 3. Results and Discussion

**Figure 1:**
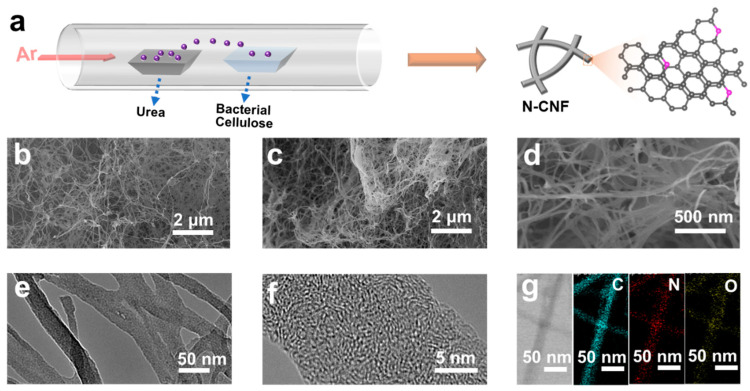
(**a**) Synthetic schematic process of N-CNF. SEM image of (**b**) CNF and (**c**) N-CNF, and (**d**) enlarged SEM image of N-CNF. (**e**) TEM image, (**f**) high-resolution TEM image and (**g**) element mapping of N-CNF.

Figure 1a shows the synthetic schematic process of N-CNF. Urea was heated in an argon atmosphere, causing the separation of the nitrogen source (including ammonia, carbamide, etc.). Under the flowing gas, the bacterial cellulose in the pyrolysis process will react with the flowing nitrogen source to obtain the N-CNF [41]. Figure 1b,c presents the SEM images of CNF and N-CNF, respectively. Both of them exhibit a nanofiber structure and are cross-linked into a conductive network. From the enlarged SEM image of N-CNF in Figure 1d, it can be seen that the nanofiber presents a diameter range of 10–100 nm. To further confirm the morphology, we provide the TEM image of N-CNF, as shown in Figure 1e. Different from the structure of other carbon nanofibers, our N-CNF presents a strong conductive cross-linked structure (similar to neuron structure), which allows for the rapid electron transfer during the potassiation–depotassiation process [42]. From the high-resolution TEM image of N-CNF in Figure 1f, a disordered lattice can be observed, indicating that our N-CNF presents an amorphous structure. Figure 1g shows the element mapping of N-CNF. It can be seen that the C, N and O elements are mainly distributed into the carbon nanofiber, indicating that the N element has been uniformly doped in N-CNF.

**Figure 2:**
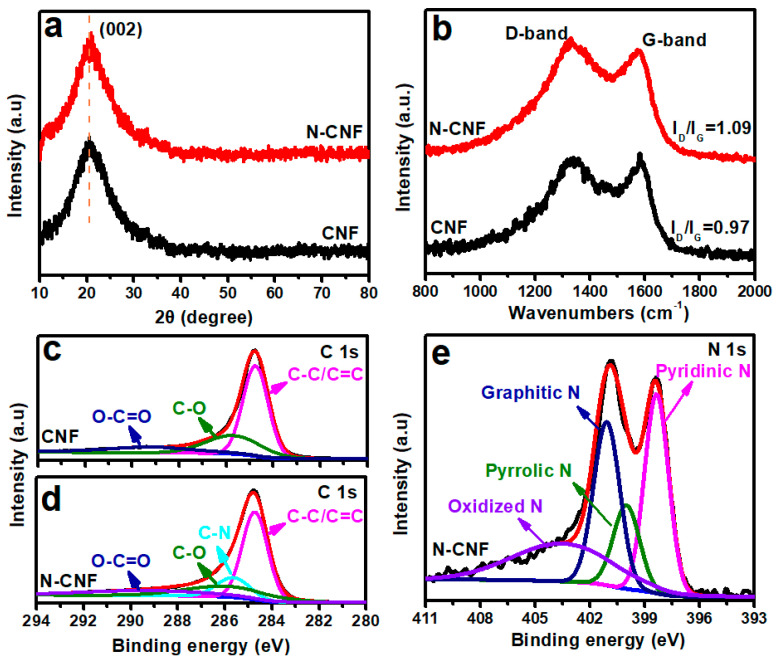
(**a**) XRD patterns and (**b**) Raman spectra of CNF and N-CNF; C 1 s XPS spectra of (**c**) CNF and (**d**) N-CNF, (**e**) N 1 s XPS spectrum of N-CNF.

Figure 2a presents the XRD patterns of CNF and N-CNF. It can be observed that both of them present a broad diffraction peak at 26°, which results from the typical amorphous carbon [43]. No obvious deviation of the diffraction peak occurred after nitrogen doping, indicating little change in the amorphous carbon lattice structure. Figure 2b shows the Raman spectra of CNF and N-CNF. Two typical peaks located at 1369 and 1617 cm^−1^ can be observed, assigned to D- and G-bands of carbon, respectively [44]. Usually, D-band indicates the amorphous structure, and G-band represents the graphitic structure of carbon. We suggest that the peak intensity ratio (*I_D_*/*I_G_*) of G- and D-bands determines as the amorphous degree of the carbon-based materials [45]. According to the calculation, the *I_D_*/*I_G_* values of CNF and N-CNF are 0.97 and 1.09, respectively, indicating that nitrogen doping can promote the amorphous structure in carbon material. Generally, the increase in amorphous structure in carbon materials contributes to the improvement of K-ion insertion [46]. To further investigate the constituents, we provide the corresponding XPS of CNF and N-CNF. According to the XPS result, the nitrogen content in N-CNF can be observed at 5.1 at%. In addition, the radical oxygen contents of CNF and N-CNF are also detected, which are 4.6 at% and 4.3 at%, respectively. Figure 2c,d presents the high-resolution C 1 s XPS spectra of CNF and N-CNF, respectively. Both of them can be fitted into three peaks at 284.8, 285.9 and 289.1 eV, corresponding to the C–C/C = C, C–O and O–C = O bonds, respectively [47]. With the nitrogen doping in carbon nanofiber, another peak at 285.6 eV can be deconvoluted, which derives from the C-N bond. In addition, we also provide the high-resolution N 1 s XPS spectrum of N-CNF to further confirm the nitrogen doping. It is found that the N 1 s XPS spectrum can be deconvoluted into four peaks at 398.4, 400.0, 401.1 and 403.5 eV, corresponding to the pyridinic N, pyrrolic N, graphitic N and oxidized-N, respectively [48,49,50,51]. Among them, it is observed that the peak at 398.4 presents more strongly than other peaks, indicating that the nitrogen doping in N-CNF mainly constitutes pyridinic N. In addition, we also provide the nitrogen adsorption–desorption isotherms and pore size distributions of CNF and N-CNF, as shown in Figure S2. According to the BET method, the specific surface areas of CNF and N-CNF can be calculated to be 79.7 and 60.1 m^2^ g^−1^, indicating that nitrogen doping in carbon nanofiber will slightly reduce the specific surface areas. From the pore size distributions of CNF and N-CNF, it is found that the main concentration region is almost constant. Generally, micropores limit the reversibility of K-ions. A significant decrease in micropore volume in N-CNF can be observed, indicating that the reversibility of N-CNF will be increased after nitrogen doping.

**Figure 3:**
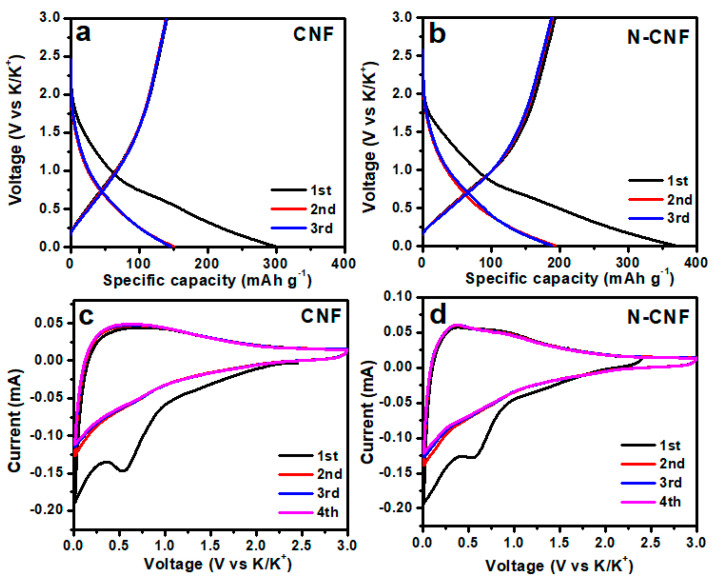
Initial GCD curves of (**a**) CNF and (**b**) N-CNF; initial CV curves of (**c**) CNF and (**d**) N-.

To investigate the electrochemical behavior of CNF and N-CNF for K-ion storage, we firstly provide their initial GCD curves for comparison (Figure 3a,b). The CNF presents an initial discharge specific capacity of 299 mAh g^−1^. After the full depotassiation process, an initial reversible specific capacity of 141 mAh g^−1^ can be achieved with an initial coulombic efficiency (CE) of 47%. After nitrogen doping, the initial discharge specific capacity of N-CNF is improved to 369 mAh g^−1^ and presents an initial reversible specific capacity of 193 mAh g^−1^, with an initial CE of 52%. The huge capacity change during the initial stage is due to the formation of a solid electrolyte interphase (SEI) layer [52]. In the subsequent cycles, the GCD curves of both electrodes almost overlap, indicating that both electrodes exhibit excellent stability for KIBs. To analyze the electrochemical reaction process of electrodes, the initial CV curves of both electrodes are also provided, as shown in Figure 3c,d. This can be observed as a typical irreversible cathodic peak of CNF at ~0.53 V in the initial cycle, attributed to the formation of the SEI layer. This result is consistent with the GCD curves. After nitrogen doping, a similar irreversible cathodic peak can be detected, but its position is slightly shifted to 0.56 V, indicating that the fractional nitrogen in N-CNF participates in the formation of the SEI layer.

**Figure 4:**
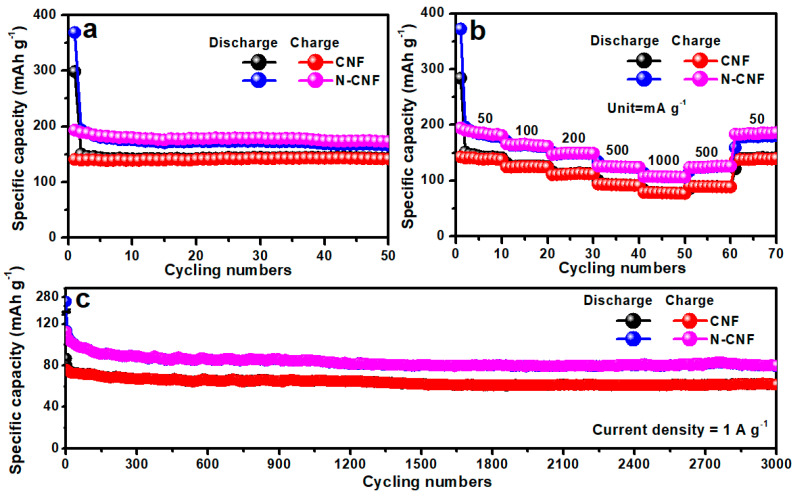
(**a**) Cycle performance, (**b**) rate performance and (**c**) long-term cycle performance of CNF and N-CNF for K-ion storage.

Figure 4a and Figure S3 show the cycling performance and CEs of CNF and N-CNF for K-ion storage. Both of the electrodes present stable cycling performance. However, the CNF electrode only exhibits a reversible specific capacity of 141 mAh g^−1^ after 50 cycles at 50 mA g^−1^. With the nitrogen doping, we observed that the electrochemical performance of N-CNF is enhanced and maintains the reversible specific capacity of 173 mAh g^−1^ after 50 cycles at 50 mA g^−1^. In addition, we also compare their rate performance for K-ion storage, as shown in Figure 4b. The CNF electrode delivers the reversible specific capacities of 141, 125, 112, 91 and 77 mAh g^−1^ at 50, 100, 200, 500 and 1000 mA g^−1^, respectively, with a capacity retention ratio of 54% (the reversible specific capacity at 1 A g^−1^: the reversible specific capacity at 50 mA g^−1^). The N-CNF electrode presents the improved reversible specific capacities of 181, 162, 149, 124 and 106 mAh g^−1^ at 50, 100, 200, 500 and 1000 mA g^−1^, respectively, with a capacity retention ratio of 58%. To investigate the improvement of rate performance for K-ion storage, we assess the EIS of electrodes, as shown in Figure S4. It is found that the N-CNF electrode presents less electrochemical impedance compared with the CNF electrode, indicating that nitrogen doping can significantly enhance the K-ion transfer ability, which will facilitate the improvement of its rate performance. To verify the cycling stability of our electrodes, the long-term cycling performances of CNF and N-CNF at a large current density of 1 A g^−1^ are provided (Figure 4c). Though the CNF electrode exhibits a stable cycling performance of 62 mAh g^−1^ after 3000 cycles, the low capacity is still inevitable. For the N-CNF electrode, the stable performance remains after nitrogen doping and the reversible specific capacity is improved to 81 mAh g^−1^ after 3000 cycles.

**Figure 5:**
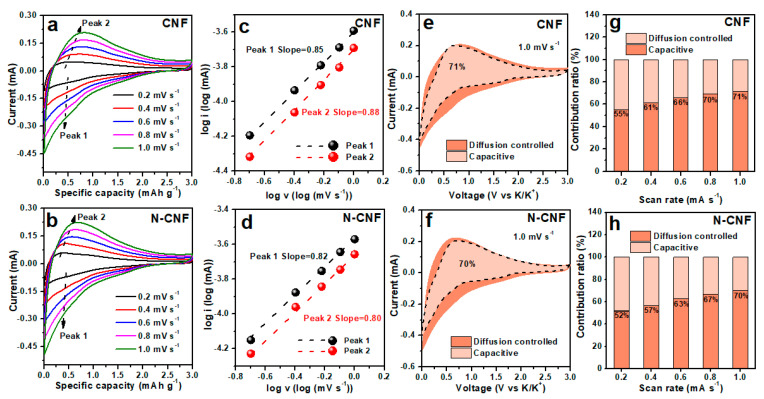
CV curves of (**a**) CNF and (**b**) N-CNF at different scan rates; relationship between log (i) and log (v) of (**c**) CNF and (**d**) N-CNF; capacitive contribution area of (**e**) CNF and (**f**) N-CNF at 1.0 mV s^−1^; capacitive contribution ratios of (**g**) CNF and (**h**) N-CNF at different scan rates.

To further demonstrate the capacity contributions, we provide the CV curves of CNF and N-CNF (Figure 5a,b). All the CV curves present a similar shape, indicating they exhibit a similar electrochemical reaction. However, it is found that the current intensity in N-CNF surpasses that of CNF greatly, which should be due to the improved K-ion storage with nitrogen doping. In addition, we also investigate their electrochemical behavior according to the change in their CV curves. The relationship of current (i) and scan rate (v) can be calculated by Equation (1) [53,54]:

(1) $$i=av^{b}$$

which can be transformed into Equation (2):

(2) logi=loga+blogv

Among them, *a* and *b* should be the adjustable parameters, and the *b* value is decided by the slope in Equation (2). Generally, a *b* value near 0.5 is derived from the diffusion-controlled behavior, and close to 1 is rooted in the capacitive behavior. It is found that all the *b* values are close to 1, indicating that both electrodes mainly present the capacitive behavior. However, the *b* values in N-CNF (Figure 5d) are lower than those of CNF (Figure 5c) because nitrogen doping can facilitate the diffusion-controlled behavior. For the quantification of their electrochemical behavior, their capacitive contributions are calculated according to Equation (3) [55]:

(3) $$i\left(V\right)=k_{1}v+k_{2}v^{1/2}$$

which can be converted to Equation (4):

(4) $$i\left(V\right)/v^{1/2}=k_{1}v^{1/2}+k_{2}$$

Among them, *k*_1_*v* and *k*_2_*v*^1/2^ are supposed to the capacitive and diffusion-controlled behaviors, respectively. According to the calculation, the N-CNF electrode receives a capacitive contribution of 70% at 1 mV s^−1^ (Figure 5f), lower than that of the CNF electrode (71%, Figure 5e). Figure 5g,h presents the capacitive contributions at different scan rates of CNF and N-CNF electrodes. The CNF electrode exhibits a capacitive contribution of 55%, 61%, 66%, 70% and 71% at 0.2, 0.4, 0.6, 0.8 and 1.0 mV s^−1^, respectively. For the N-CNF electrode, it is present at a slightly lower capacitive contribution of 52%, 57%, 63%, 67% and 70% at the scan rates of 0.2, 0.4, 0.6, 0.8 and 1.0 mV s^−1^, respectively, compared with that of the CNF electrode. This phenomenon could be due to the occurrence of a redox reaction after nitrogen doping, which increases the diffusion-controlled behaviors during the potassiation–depotassiation process.

**Figure 6:**
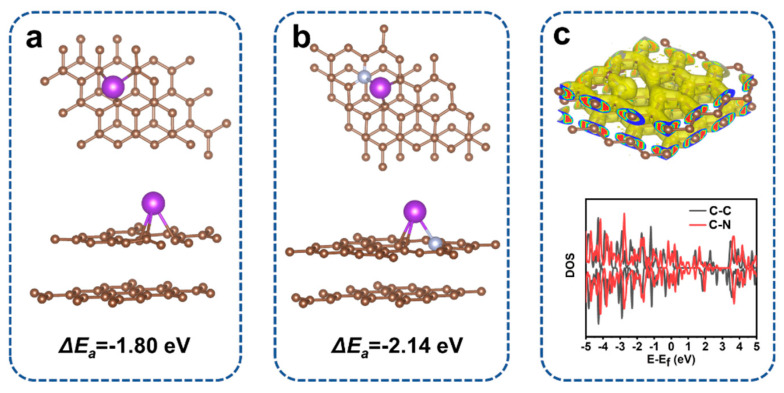
DFT calculations of the adsorption models and the electrolyte structures: (**a**) the models and energy of C after K+ absorption, (**b**) the models and energy of N–C after K+ absorption, (**c**) the electronic localization function of N–C and the density of states (DOS) for the C–C model and the C–N model.

To confirm the improvement of electrochemical performance by nitrogen doping, we also conduct the DFT calculation to obtain the relationship between nitrogen doping and the K-ion storage performance of our electrodes, as shown in Figure 6. In Figure 6a,b, we show the top and front views of the carbon layer and the nitrogen-doped carbon layer configurations after the insertion of K-ion, which are named the C–C model and C–N model, respectively. It is found that K-ion maintains the stable absorption in both configurations. According to the configurations, we calculated the *E*_2_, *E*_1_ and *µ_K_* of the C–C model and C–N models, as shown in Table S1. Therefore, the adsorption energy (∆*E_a_*) value of the C–N model is calculated to be −2.14 eV, which exhibits more negative than that of the C–C model (−1.80 eV). Due to the more negative adsorption energy, the C–N model will present a higher K atom adsorption capability [56,57]. Figure 6c shows the electronic localization function of the C–N model. It is found that nitrogen doping decorates the electronic structure of the carbon layer, which can be observed as the net gain of electronic charge between the nitrogen atom and carbon layer [58]. This phenomenon illustrates that a charge can be transferred from the nitrogen doping atom to its nearest neighboring carbon atom [58]. Furthermore, we also find that the Fermi level of the C–N model is shifted right up to the conduction band with the additional nitrogen doping according to the calculation results of the electronic density of states. It is suggested that the electronic conductivity receives a significant improvement after nitrogen doping. According to the theoretical verification, we suggest that the nitrogen doping in our carbon nanofiber not only efficiently enhances the adsorption of K-ion but also reduces the electron transmission resistance, which will remarkably facilitate the K-ion storage of our N-CNF.

In our previous work, we note that the properties of anode materials are not only related to their specific capacity but also to their voltage. Generally, the low voltage of anode materials is helpful to promote the energy density of a K-ion full battery. To better evaluate the electrochemical performance of our electrode, we calculate a new concept of relative energy density (*E_R_*) for comparison [59]. The detailed calculation formulas are as follows:

(5) $$E_{R}=\Delta UQ$$

(6) $$\Delta U=-P_{K}-V_{A}$$

(7) $$V_{A}=\int^{\;}UdQ/Q$$

**Figure 7:**
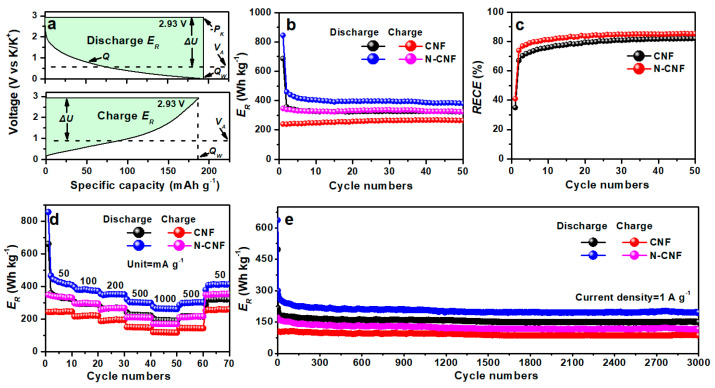
(**a**) Typical legends for the determination of the relative energy density (*E_R_*); (**b**) *E_R_* of CNF and N-CNF electrodes at the charge and discharge process, (**c**) relative energy conversion efficiency (*RECE*) of CNF and N-CNF electrodes, (**d**) rate *E_R_* performance and (**e**) long-term *E_R_* cycling performance of CNF and N-CNF electrodes.

Among them, *Q*, *V_A_*, *P_K_* and Δ*U* are specific capacity, average voltage, electrode potential of K (−2.93 V vs. K/K^+^) and the difference value of −*P_K_* and *V_A_*, respectively. To better understand the formula model, we provide a corresponding legend to illustrate them, as shown in Figure 7a. According to the calculation, the initial reversible *E_R_* of CNF is 240 Wh kg^−1^, and it maintains the *E_R_* of 256 Wh kg^−1^ after 50 cycles. With nitrogen doping, we find that the *E_R_* of N-CNF is improved. The corresponding initial reversible *E_R_* is 332 Wh g^−1^ and it maintains the reversible *E_R_* of 325 Wh kg^−1^ after 50 cycles. Furthermore, we calculate the relative energy conversion efficiency (RECEs) of CNF and N-CNF, as shown in Figure 7c, which is the ratio of charge *E_R_* and discharge *E_R_*. This value can be used to evaluate the energy conversion efficiency. It is found that the RECE of N-CNF is higher than that of CNF, which indicates that N-CNF has a higher energy conversion efficiency for K-ion storage. Figure 7d presents the rate *E_R_* of CNF and N-CNF, and it is found that N-CNF exhibits a reversible *E_R_* of 330, 294, 267, 209 and 169 Wh kg^−1^ at 50, 100, 200, 500 and 1000 mA g^−1^, respectively, which is higher than that of CNF. Moreover, the *E_R_* values of CNF and N-CNF at 1 A g^−1^ are calculated, as shown in Figure 7e. The N-CNF electrode presents an *E_R_* of 117 Wh kg^−1^, higher than that of the CNF electrode.

## 4. Conclusions

In our work, we obtained N-CNF derived from bacterial cellulose by a simple pyrolysis process, which facilitates ultra-stable K-ion storage. After nitrogen doping, our N-CNF presents a reversible specific capacity of 173 mAh g^−1^ after 50 cycles at 50 mA g^−1^. Even at a large current density of 1 A g^−1^, our electrode presents a reversible specific capacity of 81 mAh g^−1^ after 3000 cycles for KIBs, with a capacity retention ratio of 71%. To investigate the electrochemical enhancement performance of our N-CNF, we provide the calculation results according to DFT calculation, demonstrating that nitrogen doping in carbon favors K atom adsorption during the potassiation process. This behavior will contribute to the enhancement of electrochemical performance for KIBs. In addition, we noticed that our electrode exhibited a low voltage plateau during the potassiation–depotassiation process. To further evaluate this performance, we calculate the “relative energy density” for comparison, and the results illustrate that our electrode presents a high “relative energy density”, indicating that our N-CNF is a promising anode material for KIBs.

## Supplementary Materials

The following are available online at https://www.mdpi.com/article/10.3390/nano11051130/s1, Figure S1: (a) SEM, (b) TEM and (c) high-resolution TEM image of CNF; Figure S2: (a) nitrogen adsorption-desorption isotherms and (b) pore diameter distribution of CNF and N-CNF; Figure S3: CEs of CNF and N-CNF; Figure S4: EIS of CNF and N-CNF after 50 cycles; Table S1: The obtained parameters based on DFT calculation.
