# Supplementary material for: Ultra-Stable Potassium Ion Storage of Nitrogen-Doped Carbon Nanofiber Derived from Bacterial Cellulose
Source: Nanomaterials (Basel). 2021 Apr 27;11(5):1130. doi: 10.3390/nano11051130 (PMC8145622; doi:10.3390/nano11051130)
Supplement: Supplementary file 1 [file nanomaterials-11-01130-s001.zip › nanomaterials-1195452-supplementary.pdf]

nanomaterials

Supplementary material

Ultra-Stable Potassium Ion Storage of Nitrogen-Doped Carbon Nanofiber Derived from Bacterial Cellulose

Liang Ma [1,2,†], Jinliang Li [2,†], Zhibin Li [2], Yingying Ji [2], Wenjie Mai [2] and Hao Wang [1,*]

1 Guangdong Provincial Key Laboratory of Micro/Nano Optomechatronics Engineering, College of Mechatronics and Control Engineering, Shenzhen University, Shenzhen 518060, China; maliang2415@jnu.edu.cn (L. M.)

2 Siyuan Laboratory, Guangdong Provincial Engineering Technology Research Center of Vacuum Coating Technologies and New Materials, Department of Physics, Jinan University, Guangzhou, Guangdong 510632, China; lijinliang@email.jnu.edu.cn (J.L.); pibetaguita@gmail.com (Z.L.); jiyingying07@163.com (Y.J.); wenjiemai@email.jnu.edu.cn (W.M.);

* Correspondence: whao@szu.edu.cn

† These authors contributed equally.

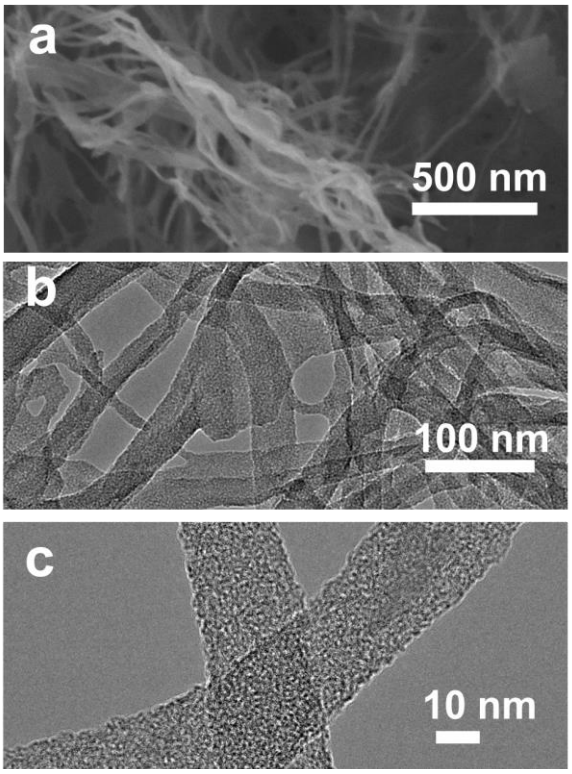

Figure S1. (**a**) SEM, (**b**) TEM and (**c**) high-resolution TEM image of CNF.

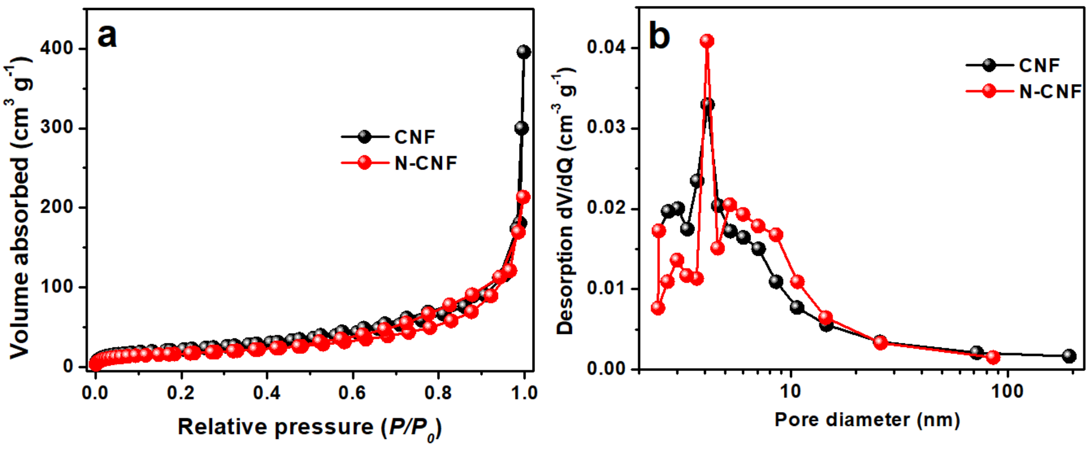

Figure S2. (**a**) nitrogen adsorption-desorption isotherms and (**b**) pore diameter distribution of CNF and N-CNF.

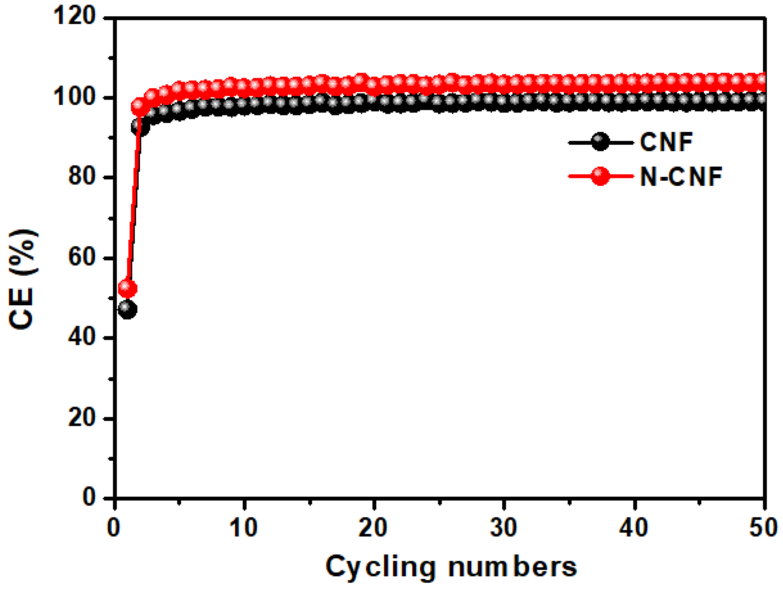

Figure S3. CEs of CNF and N-CNF.

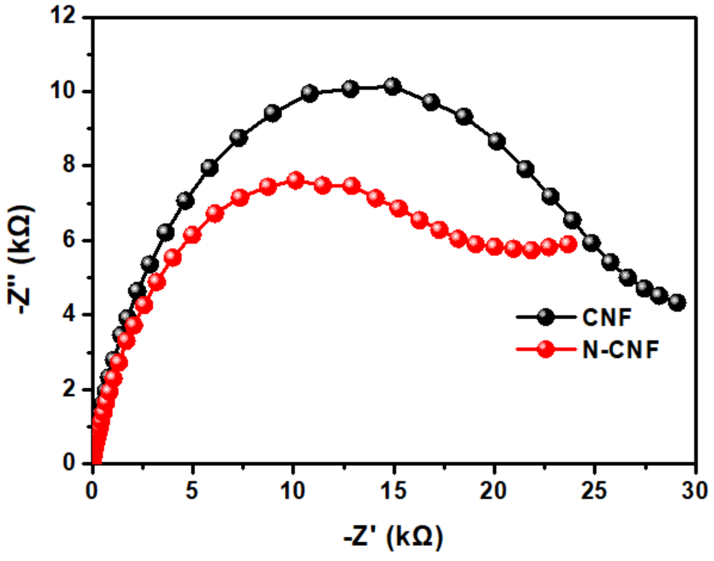

Figure S4. EIS of CNF and N-CNF after 50 cycles.

Table S1. The obtained parameters based on DFT calculation.

System	E_2 (eV)	E_1 (eV)	μK (eV)	ΔE_a (eV)
C-C model	-318.886	-316.785	-0.304	-1.80
C-N model	-320.046	-317.604	-0.304	-2.14
